# Efficacy and safety of endovenous laser ablation with the 1470 nm diode laser using a novel optical probe

**DOI:** 10.7150/ijms.70916

**Published:** 2022-03-28

**Authors:** Karsten Hartmann, Patrick Gholam, Carmen Dietrich, Christine Fink

**Affiliations:** 1 Venenzentrum Freiburg, Zähringer Str. 14, 79108, Freiburg, Germany.; 2 Department of Dermatology, University of Heidelberg, Heidelberg, Germany.

**Keywords:** Varicose veins, EVLA, Laser ablation, Efficacy, Safety, Outcome

## Abstract

**Objectives:** Outcome assessment of a novel optical fiber probe for the 1470 nm diode laser under real-world conditions.

**Methods:** Prospective clinical pilot study in 10 patients undergoing endovenous laser ablation with a follow-up period of 1 year. Primary endpoints were efficacy and safety. Secondary endpoints include, inter alia, quality of life and patient satisfaction.

**Results:** After a follow-up period of 1 year all treated vein segments were still occluded. Only mild and short-term side effects (hematoma, ecchymosis and hyperpigmentation) were observed. No intake of pain medication was needed and a quick return to normal activity was documented (0.9 days). Clinical hallmarks of the venous disease (VCSS) improved significantly (p= .003). All patients were very satisfied with the treatment and quality of life (AVVQ) was significantly improved after the procedure (p=.008).

**Conclusions:** The study demonstrates that the endoluminal treatment with the novel fiber probe is highly effective and safe.

## Introduction

**Figure 1:**
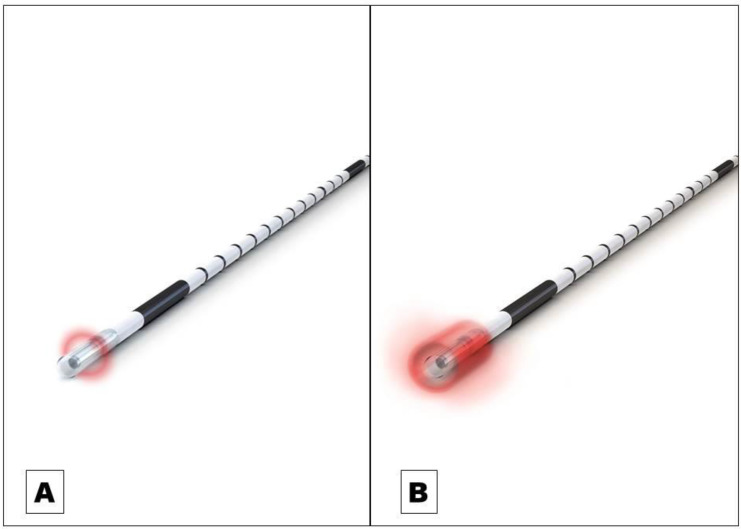
** A** Commonly used optical fiber probe with radial light emission profile with energy release in a well-defined narrow area of approximately 0.5 mm in length along the vein (Example shows “neoLaser CORONA 360 Fused Fiber”, manufacturer: Light Guide Optics International Ltd.) **B** The newly developed optical fiber probe has a much wider emission profile. In this way a large-scale and more even irradiation of the vein wall can be achieved. (Example shows “neoLaser CORONA Infinite Ring Fiber”, manufacturer: Light Guide Optics International Ltd. Image courtesy of Light Guide Optics Germany GmbH, Germany)

Endovenous laser ablation (EVLA) is a safe endothermal treatment option for incompetent varicose veins of the lower extremities. In EVLA, a diode laser fiber is inserted percutaneously into the varicose vein. Permanent vein occlusion is caused by laser-induced thermal damage of the endothelium followed by subsequent fibrosis 1,2,3. Since the first report on EVLA was published in 1999 a substantial further development of the technology took place 4. Treatment efficacy (i.e. the occlusion rate) and safety strongly depends on how the laser energy is dosed within the treated vein. The majority of the current established optical fiber probes emit the laser radiation radially onto the vein wall 5. The release of laser energy is typically confined to a well-defined narrow area of approximately 0.5 mm in length along the vein (Figure 1A). To treat a vein segment over its full length, it is therefore necessary to move the probe through the vein while the laser is activated 2,5. Endovenous energy distribution with subsequent heating of the vein wall should be as uniform as possible. On one hand, unintentional local underdosing of energy leads to a decreased occlusion rate; but on the other hand, an overdose of radiation is accompanied with an increased complication rate (i.e. venous puncture with bruising, damage to surrounding nerves). In this context, a larger, more even energy distribution of the laser radiation might be advantageous. Within this study we evaluated an innovative optical fiber probe with a wider emission profile. In contrast to previous probes the laser energy is emitted evenly over a larger area of approx. 4 mm, so that a uniform radiation is possible (Figure 1B). In this way a large-scale and more even irradiation of the vein wall can be achieved which might have an impact on the safety and efficacy of EVLA. Aim of this clinical study was the evaluation of the safety and efficacy of the new optical fiber probe under “real world” conditions in a prospective setting with a one year-follow-up.

## Methods

This clinical study was performed in a prospective setting in ten consecutive patients (with eleven insufficient great saphenous veins (GSV)) with a medical indication for elective thermal ablation. Written informed consent for the procedure was given by all patients. The study was conducted in accordance with the Declaration of Helsinki principles (2013) and applicable local government regulations and independent Ethics Committee policies and procedures (ethics approval number F-2019-122).

### Study endpoints

The primary objective of this study was to assess the efficacy (occlusion rate) and safety (intraoperative and postoperative complications e.g. deep vein thrombosis, paresthesia) of the new optical fiber probe within a follow-up period of one year. Secondary objectives of this study include, inter alia, postoperative pain, absence from work and normal activity, disease-specific quality of life and patient satisfaction. In total, six study visits were performed. Patients were examined at the time of recruitment (Baseline Visit; V1), the day of EVLA (V2), within ten days after EVLA (V3), two months (V4), six months (V5) and one year (V6) postoperatively. All patients were examined clinically and by duplex ultrasound by an experienced phlebologist. Postoperative pain was assessed by means of the VAS score (visual analogue pain scale 6). Patients were asked to evaluate the pain on a scale of one (no pain) to ten (severe pain) within ten days after EVLA in four categories: the greatest pain since the last visit; currently experienced pain in the area of the operated limb; the current pressure pain; the most severe pressure pain since the last visit. Moreover, postoperative pain (in days), intake of pain medication and absence from work and normal activity (in days) was documented. Furthermore, each patient was required to evaluate satisfaction with the endoluminal treatment on a scale of one (very satisfied) to five (very unsatisfied) after two months, six months and one year. The disease severity and outcome of therapy for venous disease was assessed via validated Venous Clinical Severity Score (VCSS) 7. The VCSS facilitates features of venous disease that change with treatment and includes ten hallmarks of venous disease. Disease-specific quality of life was determined by means of the Aberdeen Varicose Vein Symptom Severity Score (AVVQ) which is a validated 13-question survey addressing all elements of varicose vein disease 8. Both questionnaires are scored from 0 (indicating no effect on the patient from varicose veins) to 100 (indicating severe effect).

### Endovenous procedure

In all patients EVLA was performed with the novel market approved optical fiber probe “neoLaser CORONA Infinite Ring Fiber” (manufacturer: Light Guide Optics International Ltd.) suitable for the 1470 nm diode laser (“neoV1470”, manufacturer: neoLaser, Israel) according to the manufacturer's instructions. The laser fiber was introduced to the GSV at the distal point of insufficiency via a sheath followed by the positioning at the sapheno-femoral junction (SFJ) under sonographic control. The entire EVLA was performed under sonographic monitoring and tumescent local anesthesia. Laser energy was delivered at 10W. Additionally, foam sclerotherapy was performed after EVLA when insufficient tributaries were present which was determined by clinical examination and by duplex ultrasound. Taking into account possible contraindications thrombosis prophylaxis with low molecular weight heparins (40 mg s.c.) was given immediately after EVLA for one day and compression therapy with class II stockings was recommended for ten days.

### Statistical analysis

Descriptive analyses were performed (frequency, mean, range). Non-parametric tests were applied to assess for statistical significance (Wilcoxon signed rank). p<0.05 was considered statistically significant. SPSS Version 25 (IBM, SPSS; Chicago, Illinois, USA) was used.

### Patient and Public involvement

Patients or the public were not involved in the design, or conduct, or reporting, or dissemination plans of our research.

## Results

### Patients and GSV characteristics

Ten patients with eleven insufficient GSV (n=11) were recruited between January 2020 and May 2020. A reflux time of >0.5 seconds for GSV was used to diagnose the presence of reflux. Three (27.3%) of the patients' legs showed CEAP stage 2 with varicose veins (with a diameter of 3mm or more), five (45.5%) legs showed CEAP stage 3 with edema and three (27.3%) legs showed CEAP stage 4 with changes in skin and subcutaneous tissue secondary to chronic venous disorder. All patients showed a complete incompetence of the GSV. The reflux passed directly through the saphenofemoral junction (SFJ) into the GSV due to an incompetent terminal and preterminal valve. One leg (9.1%) showed Hach stage II with an insufficiency from the SFJ to a hand's breadth above the knee joint. The majorities of the GSV showed Hach stage III (90.9%) with an insufficiency starting from the SFJ to below the knee. Mean diameter of the GSV at the SFJ was 8 mm, mean diameter 3 cm below SFJ was 7.3 mm and mean diameter 15 cm below SFJ was 6.3 mm. Mean length of the treated GSV was 43.9 cm. The average LEED for GSV ablation was 69.75 J/cm. In 90.9% EVLA was combined with foam sclerotherapy in tributaries. Foam sclerotherapy was performed directly after EVLA. Mean dose of administered foam was 3,3 ml (min-max: 1-6 ml) (Table 1).

### Efficacy and Safety

A duplex ultrasound was performed by an experienced phlebologist at six time points (before endovenous laser ablation (EVLA), at the day of the procedure, within ten days after EVLA, two months, six months and one year after EVLA. Additionally, a clinical examination was performed at each of the six time points. Intraoperatively no complications occurred. Within ten days after EVLA two hematomas and one ecchymosis were present which were dissolved until the next examination. Examination after two and six months showed a hyperpigmentation in one patient's leg in the treated area which was gone at the next follow-up visit. Deep vein thrombosis, pulmonary embolism, phlebitis, paresthesia or recurrent varicose veins were not seen in any of the patients. Sonography within ten days after EVLA showed an occlusion rate of 100%. After one year of follow-up all treated vein segments were still occluded (distal point of insufficiency until SFJ) in all patients (Table 2).

### Post-operative pain and absence from work

Data analysis showed that patients experienced only mild postoperative pain with an average duration of 0.9 days. None of the patients had to take pain medication after EVLA. The average number of absence from work and normal activity in days was 0.8 (Table 3).

### Patient-reported outcome measures: response to therapy (VCSS), quality of life (AVVQ) and patient satisfaction

**Figure 2:**
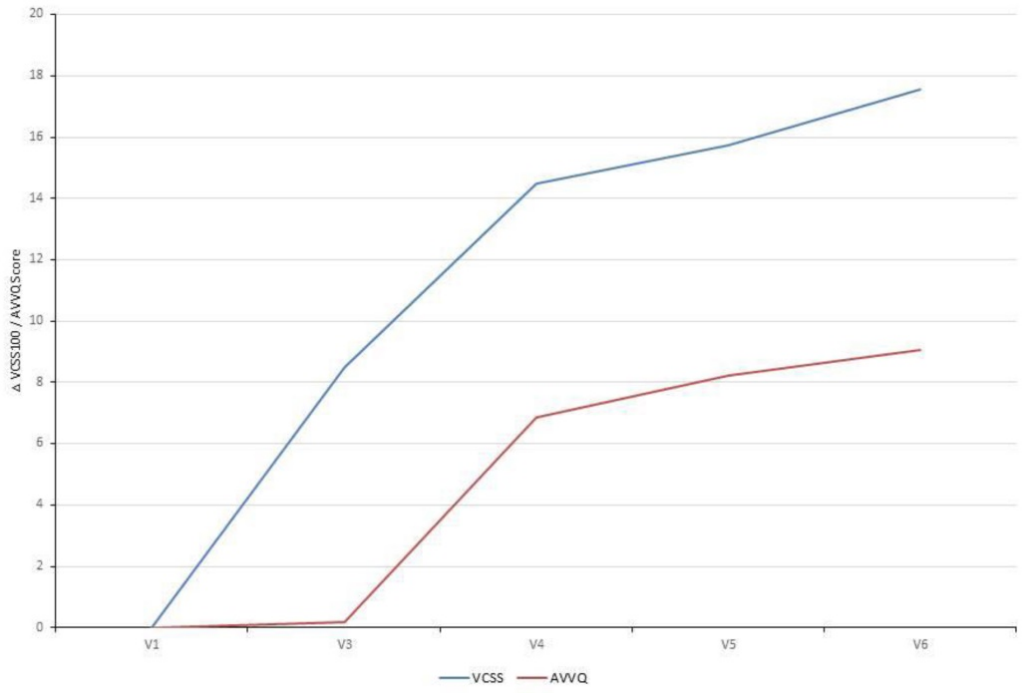
Patient-reported therapy outcome (VCSS) and quality of life (AVVQ): Analysis of the VCSS questionnaire showed a significant improvement over time. The disease-specific quality of life (AVVQ) was significantly improved two months after EVLA. Absolute difference of each study visit correlated to the Baseline Visit (VCSS: mean 18.48 and AVVQ: mean 10.87) is depicted.

The VCSS questionnaire includes ten clinical hallmarks of venous disease (e.g. pain, edema, inflammation, number of active ulcers) that change with treatment and therefore reflects changes in response to therapy. Already ten days after EVLA the VCSS showed a significant improvement which continued to improve over time when correlated to the Baseline Visit (V1). The disease-specific quality of life (AVVQ) was significantly improved two months after EVLA. Furthermore, evaluation of patient satisfaction showed that all patients were very satisfied with the treatment (Table 4, Fig. 2).

## Discussion

Over the last decades tremendous improvements could be observed in the treatment of varicose veins. In endovenous laser ablation (EVLA) permanent vein occlusion is caused by laser-induced thermal damage of the endothelium followed by subsequent fibrosis 1-3. Constantly new technologies and products are developed and gain market approval 1-3. Currently the 1470nm diode laser with the radial fiber probe is widely used for thermal ablation of the GSV which was shown to be highly effective and safe 5. Nevertheless, a continuous improvement of the technology should be targeted with an even higher occlusion rate and increased safety.

Within this prospective clinical study an innovative optical fiber probe was evaluated for the first time under “real world” conditions. The assessed fiber probe is characterized by a larger and more even energy distribution of the laser radiation. In comparison to the radial fiber probe the laser energy is emitted evenly over a larger area which allows for a more uniform radiation. In this study a total of eleven insufficient GSV were occluded and patients were regularly followed up over a period of one year.

Primary endpoints of this study were to assess the efficacy and safety of the fiber probe. Efficacy reflected by the occlusion rate was 100% within ten days after EVLA. After one year of follow-up all treated vein segments were still occluded. Furthermore, data analysis showed no intraoperative complications. Postoperatively, only mild side effects were observed (two hematomas, one ecchymosis and one hyperpigmentation which were dissolved until the next visit). Moderate or severe adverse events (e.g. deep vein thrombosis, superficial phlebitis and paresthesia) were not seen in the study population.

Secondary study objectives included post-operative pain and absence from work and patient satisfaction. On average patients experienced only mild postoperative pain with a short mean duration of 0.9 days. None of the patients had to take pain medication after EVLA. Patients were able to return quickly to work and normal activity after a mean duration of sick leave of 0.8 days.

Since the inclusion of patients' preferences and needs is a fundamental requirement for a successful physician-patient relationship, the assessment of patient-reported outcome measures in clinical trials is essential. Therefore, this study evaluated as further secondary study objectives the patient-reported outcome measures patient satisfaction, the disease severity and outcome of therapy for venous disease (by means of the VCSS questionnaire) and the disease-specific quality of life (by means of the AVVQ questionnaire). Evaluation of patient satisfaction showed that all patients were very satisfied with the treatment two months, six months and one year after EVLA. The VCSS questionnaire reflects changes in response to EVLA and showed a significant improvement right after the procedure. Clinical hallmarks of the venous disease further improved significantly over the follow-up period of one year. Also the disease-specific quality of life was significantly improved two months after EVLA.

Altogether, our study reveals several limitations. The study findings need to be interpreted in the context of the study design and the patient population. Since this is a pilot study the relatively small sample size might limit the generalizability of the results. Therefore, the next step is to evaluate the novel fiber probe in a randomized controlled setting in comparison to the radial fiber probe with a bigger sample size for more representative results. Secondly, a longer follow-up period might provide further important long-term information concerning the occlusion rate. Strengths of the study are that the patients were observed in a prospective setting under real-life clinical conditions. Additionally, essential patient related outcome measures (e.g. quality of life and response to therapy) were assessed by validated scores.

In conclusion this study demonstrates that the 1470nm diode laser with the novel fiber probe is a highly effective technical innovation with an occlusion rate of 100% after a follow-up of one year. The data demonstrates the excellent safety with only few and mild complications.

## Figures and Tables

**Table 1:** Patients and GSV characteristics are depicted.

Patients (No.)	10
Legs (No.)	11
Age in years (mean; range)	53.2 (28-76)
Gender (male/female)	6/4
Height (cm) (mean; range)	172 (153-186)
Weight (kg) (mean; range)	74.9 (59-104)
Body side (left/right)	9/2
Hach stage (No. (%))	
Hach II	1 (9.1%)
Hach III	10 (90.9%)
CEAP stage (No. (%))	
C2	3 (27.3%)
C3	5 (45.5%)
C4	3 (27.3%)
GSV Diameter (mm) (mean; range)	
SFJ	8 mm (5.2-10.9)
3 cm below SFJ	7.3 mm (5.3-11.1)
15 cm below SFJ	6.3 mm (5-9.7)
LEED (J/cm) (mean; range)	69.75 (49-95.6)
Length of the treated GSV (cm) (mean; range)	43.9 (21-59)
Combined therapy* (No. (%))	10 (90.9%)

CEAP: clinical, aetiological, anatomical and pathological; GSV: great saphenous vein; SFJ: sapheno-femoral junction, LEED: linear endovenous energy density; *Combined therapy = EVLA plus foam sclerotherapy in tributaries.

**Table 2:** Intra- and postoperative complications and occlusion rate

Complications (No.)	V2 (EVLA)	V3 (10 d)*	V4 (2 m)*	V5 (6 m)*	V6 (1 yr)*
Hematoma	0	2	0	0	0
Ecchymosis	0	1	0	0	0
Hyperpigmentation	0	0	1	1	0
Deep vein thrombosis	0	0	0	0	0
Pulmonary embolism	0	0	0	0	0
Phlebitis	0	0	0	0	0
Paresthesia	0	0	0	0	0
Recurrent varicose veins	0	0	0	0	0
No complications (No.)	11	8	10	10	11
GSV occlusion (No.)	11	11	11	11	11

*Follow-up visits were performed at the day of EVLA (V2), within 10 days after EVLA (V3), 2 months (V4), 6 months (V5) and 1 year (V6) postoperatively. GSV: great saphenous vein

**Table 3:** Post-operative pain and absence from work

Post-operative pain intensity within 10 days after EVLA (0-10) (mean±SD)	
The greatest pain since the last visit	2.78 ±2.59
Currently experienced pain in the area of the operated limb	1.22 ±0.67
The most severe pressure pain since the last visit	3.33 ±2.78
The current pressure pain	1.89 ±1.05
Postoperative pain in days (mean; range)	0.9 (0-3)
Intake of pain medication in days (mean; range)	0.0 (0-0)
Absence from work and normal activity in days (mean; range)	0.8 (0-4)

SD: standard deviation

**Table 4:** Disease severity and outcome of therapy for venous disease (VCSS), disease-specific quality of life (AVVQ) and patient satisfaction

VCSS (0-100)	(mean±SD)	p-value*
V1 (Baseline)	18.48 (±10.99)	
V3 (10 days)	10 (±3.94)	.034
V4 (2 months)	4 (±4.66)	.007
V5 (6 months)	2.73 (±5.12)	.003
V6 (1 year)	0.91 (±1.56)	.003
**AVVQ (0-100)**	**(mean±SD)**	p-value*
V1 (Baseline)	10.87 (±7.15)	
V3 (10 days)	10.69 (±6.65)	.906
V4 (2 months)	4.01 (±4.79)	.025
V5 (6 months)	2.66 (±3.59)	.012
V6 (1 year)	1.82 (±3.37)	.008
**Patient satisfaction (1-5) (mean; range)**		
V4 (2 months)	1 (1-1)	
V5 (6 months)	1.1 (1-2)	
V6 (1 year)	1 (1-1)	

VCSS: Venous Clinical Severity Score; AVVQ: Aberdeen Varicose Vein Symptom Severity Score; SD: standard deviation; *p-values: V3, V4, V5, V6 were correlated to V1.

## Abbreviations

EVLA: Endovenous Laser Ablation
GSV: Great Saphenous Vein
SFJ: Sapheno-femoral junction
VAS: visual analogue pain
VCSS: Venous Clinical Severity Score
AVVQ: Aberdeen Varicose Vein Symptom Severity Score
ICH-GCP: International Conference on Harmonization-Good Clinical Practice
CEAP: Clinical, aetiological, anatomical and pathological
SD: Standard Deviation
